# Enhancement of Semen Cryopreservation from Native Thai Bulls Through *Moringa oleifera* Leaf Extract Supplementation

**DOI:** 10.3390/ani15030439

**Published:** 2025-02-05

**Authors:** Supakorn Authaida, Wuttigrai Boonkum, Vibuntita Chankitisakul

**Affiliations:** 1 Department of Animal Science, Faculty of Agriculture, Khon Kaen University, Khon Kaen 40002, Thailand; supakorn.u@kkumail.com (S.A.); wuttbo@kku.ac.th (W.B.); 2 The Research and Development Network Center of Animal Breeding and Omics, Khon Kaen University, Khon Kaen 40002, Thailand

**Keywords:** *Moringa*, freezing semen, antioxidant, lipid peroxidation, Thai native bull

## Abstract

Cryopreservation of native Thai bull semen often results in significant post-thaw quality reduction, underscoring the need for effective cryoprotective strategies. This study investigated the effect of *Moringa oleifera* leaf extract (MOLE) as an antioxidant supplementation by incorporating four MOLE concentrations (0–1.5% [*w*/*v*]) into a standard semen extender, followed by cryopreservation using liquid nitrogen vapor freezing. Data were analyzed using a randomized complete block design with Tukey’s post hoc test (*p* < 0.05). Post-thaw analysis of semen revealed that 1 mg/mL MOLE significantly enhanced total sperm motility, progressive sperm motility, sperm viability, and sperm plasma membrane integrity compared to the control and other MOLE concentrations (*p* < 0.05). This concentration also improved the amplitude of lateral head displacement and curvilinear velocity and reduced malondialdehyde levels in semen samples (*p* < 0.05), indicating reduced lipid peroxidation. Higher MOLE concentrations negatively impacted semen quality. In conclusion, supplementation with 1 mg/mL MOLE markedly improved post-thaw semen quality and reduced lipid peroxidation, suggesting its potential as an antioxidant for enhancing reproductive outcomes in native Thai bulls.

## Simple Summary

Cryopreservation of bull semen often reduces post-thaw quality. This study investigated the potential of *Moringa oleifera* leaf extract (MOLE) supplementation in semen extenders to improve the cryopreservation of semen from native Thai bulls. While MOLE exhibited significant antioxidant activity, only a concentration of 1 mg/mL improved post-thaw sperm motility, viability, and membrane integrity, reducing oxidative stress. Higher concentrations were detrimental. These results suggest that MOLE, at an optimized concentration, may enhance semen cryopreservation and fertility in native Thai bulls.

## 1. Introduction

Cryopreservation is crucial for preserving the genetic diversity of livestock, particularly native breeds like Thai bulls, which are vital for biodiversity and local adaptation. However, cryopreservation exposes semen to substantial oxidative stress and cellular damage, often reducing post-thaw sperm quality and fertility [1]. These challenges may be particularly pronounced in native Thai bulls, as tropical conditions, including elevated temperatures, exacerbate oxidative damage by increasing lipid peroxidation and reducing antioxidant enzyme activity, and thus, negatively impacting semen cryopreservation [2,3]. This necessitates effective interventions to enhance the cryopreservation process and protect sperm viability in native Thai bulls.

A promising approach to address oxidative stress during cryopreservation is the incorporation of antioxidants into semen extenders. Antioxidants neutralize reactive oxygen species (ROS), reduce lipid peroxidation, and stabilize sperm membranes, thereby improving post-thaw semen quality [4]. Various supplements have been evaluated in recent years, including both synthetic and natural antioxidants. Synthetic antioxidants such as glutathione, cysteine, taurine, and butylated hydroxytoluene have demonstrated efficacy in mitigating oxidative damage [5,6,7]. However, concerns regarding toxicity, biocompatibility, and costs have driven interest in natural antioxidants derived from plants.

Among natural antioxidants, polyphenol- and flavonoid-rich plant extracts have received significant attention due to their strong antioxidative properties [8]. For instance, supplementation with extracts from green tea (*Camellia sinensis*), rosemary (*Rosmarinus officinalis*), and turmeric (*Curcuma longa*) has been shown to enhance sperm motility, membrane integrity, and viability in various species [8]. Similarly, studies have demonstrated the benefits of using *Moringa oleifera* leaf extract (MOLE) as a natural antioxidant for cryopreservation [9,10,11]. *Moringa oleifera*, commonly known as the drumstick tree, is a nutritionally dense plant rich in bioactive compounds such as flavonoids, carotenoids, and phenolic acids [12]. These compounds exhibit strong antioxidant activity and have been linked to improved reproductive health and reduced oxidative stress [12,13,14,15].

MOLE has shown promise in enhancing semen cryopreservation by reducing oxidative stress across various species. For example, supplementation with MOLE at concentrations of 400 µg/mL improved the post-thaw quality of goat semen [9], while optimal concentrations for buffalo bull semen were reported to be 600 µg/mL and 1500 µg/mL [10,11]. These findings highlight the importance of species-specific optimization of MOLE supplementation to achieve the best cryopreservation outcomes. Despite these advancements, the effect of MOLE on the cryopreservation of native Thai bull semen has not been explored, leaving a critical gap in the literature.

This study aims to investigate the efficacy of various MOLE concentrations added to a semen extender for cryopreserving the semen of native Thai bulls. Post-thaw semen quality was assessed together with malondialdehyde (MDA) concentration, a marker of lipid peroxidation, with the goal of identifying an optimal MOLE concentration for protecting sperm viability and functionality.

## 2. Materials and Methods

The experimental procedures were reviewed and approved by the Ethics Committee on Animal Experimentation of the National Research Council of Thailand (Record no. IACUC-KKU 133/67; Reference no. 660201.2.11/888 (154)).

MOLE was sourced from AP Operations Co., Ltd., Chonburi, Thailand. Unless otherwise specified, all the other chemicals were purchased from Sigma-Aldrich (St. Louis, MO, USA).

### 2.1. Quantitative Determination of Antioxidants in MOLE

The antioxidant properties of MOLE were assessed by measuring the total phenolic content (TPC) and 2,2-diphenyl-1-picrylhydrazyl (DPPH) radical scavenging activity, as previously described [16].

TPC was quantified using the Folin–Ciocalteu (F-C) assay. Briefly, a 10 mg/mL MOLE solution and gallic acid standards (12.5–400 µg/mL) were prepared. Reaction mixtures (50 µL sample or standard + 250 µL F-C reagent + 100 µL 75% Na_2_CO_3_) were incubated for 30 min at room temperature. Absorbance was measured at 765 nm using a microplate reader (TECAN, infinite^®^ 200 PRO, Tecan Group Ltd., Mannedorf, Switzerland). TPC was calculated from a gallic acid calibration curve and expressed as mg gallic acid equivalents (GAE)/g of dry MOLE.

The antioxidant capacity of MOLE was evaluated using the DPPH assay. A 100 µL 0.20 mM DPPH solution (prepared in ethanol) was mixed with 100 µL MOLE solution in a 96-well plate and incubated in the dark for 30 min at room temperature. Absorbance was measured at 517 nm using a microplate reader. Ascorbic acid was used as a positive control. The DPPH scavenging activity (%) was calculated as follows: [(AbsorbanceDPPH − AbsorbanceMOLE + DPPH)/AbsorbanceDPPH] × 100. The IC_50_ value (the concentration required to achieve 50% inhibition) was determined from the scavenging activity versus MOLE concentration plot.

### 2.2. Semen Collection

Semen samples were collected from six native Thai bulls (3–4 years old) housed individually in an open-house system at the beef cattle farm, Department of Animal Science, Faculty of Agriculture, Khon Kaen University, Thailand (average temperature, 27.23 °C; relative humidity, 83%). Each bull was fed a daily diet of 3 kg of concentrated feed (14% protein) and 15 kg of rice straw, with water provided ad libitum. Semen was collected twice weekly using an electroejaculator (ElectroJac IV; Neogen, Lexington, KY, USA) and transported to the laboratory at 37 °C for immediate evaluation. Over the study period, this resulted in a total of 24 ejaculations.

Ejaculate volume was measured using a 10 mL syringe (accuracy ± 0.05 mL). Motility was assessed within 30 min of collection using phase-contrast microscopy (400× magnification), with samples maintained at 37 °C on a thermostatically controlled plate. Sperm viability was evaluated via eosin-nigrosin staining [17]. Sperm concentration was determined using a hemocytometer following dilution (1:200) with 4% sodium chloride solution to immobilize the sperm. Only ejaculates meeting the criteria of ≥85% total motility and a sperm concentration of ≥400 × 10^6^ sperm/mL were used for cryopreservation.

### 2.3. Semen Extender

A Tris-egg yolk extender was prepared with the following composition per 100 mL of ultrapure water: 20% (*vol*/*vol*) egg yolk, 1.675 g citric acid monohydrate, 1.250 g fructose, 3.028 g Tris base, 0.100 g streptomycin, and 0.06 g penicillin. Different concentrations of MOLE (0, 0.5, 1, and 1.5 mg/mL) were added to aliquots of the extender. For each MOLE concentration, two subgroups were created: one containing 14% glycerol and the other without glycerol.

### 2.4. Semen Cryopreservation

Fresh semen samples were divided into four equal aliquots and diluted at 30 °C with the Tris-based extender (control) or Tris-based extender supplemented with MOLE (0.5, 1, and 1.5 mg/mL) to achieve an initial concentration of 80 × 10^6^ sperm/mL. The diluted semen was transferred to pre-warmed tubes and cooled gradually from 37 °C to 5 °C over 2 h in a refrigerator (TOSHIBA, Toshiba Thailand Co., Ltd., Bangkok, Thailand). Subsequently, an equal volume (1:1) of extender containing 14% glycerol was added, resulting in a final concentration of 40 × 10^6^ sperm/mL before loading into straws, followed by a 3 h equilibration period at 5 °C. Following this cooling process, sperm quality was evaluated by assessing total motility, ensuring that the total motility of all groups exceeded 70% prior to the freezing procedure. Subsequently, the cooled semen was loaded into 0.5 mL polyvinyl chloride straws (IMV Technologies, L’Aigle, France) to achieve a final concentration of 80 × 10^6^ sperm/mL. The straws were frozen using a controlled-rate freezing protocol. They were initially placed 4 cm above the surface of liquid nitrogen (7 L) in a Styrofoam box (25 × 35 × 30 cm) for 15 min and then directly immersed into liquid nitrogen (−196 °C) for storage (at least 1 month). For semen evaluation, three straws per treatment per cow were thawed in a 37 °C water bath for 30 s.

### 2.5. Evaluation of Post-Thaw Sperm Quality

Post-thaw sperm quality was analyzed using computer-assisted sperm analysis (CASA; Olympus software version 10, HIM-IVOS; Hamilton Thorne Biosciences, Beverly, MA, USA). The following parameters were assessed: total motility (MOT), progressive motility (PMOT), average path velocity (VAP), straight-line velocity (VSL), curvilinear velocity (VCL), amplitude of lateral head displacement (ALH), beat cross frequency (BCF), straightness (STR), and linearity (LIN). For the assessment, 5 µL aliquots of diluted semen were placed on separate slides (maintained at 37 °C). Five microscopic fields per slide (more than 300 sperm) were captured for 10 s using an Olympus 10× phase-contrast lens and DP71/25 digital camera at 30 frames/s (fps; 60 Hz). Sperm with VAP < 5 µm/s were considered immotile, whereas those with VAP > 20 µm/s and STR ≥ 80% were classified as progressively motile.

Sperm viability was assessed using eosin-nigrosin staining [17]. A 5 µL semen sample was mixed with 10 µL of eosin-nigrosin stain, smeared onto a slide, air-dried, and examined microscopically (Olympus CH30, Tokyo, Japan; 400× magnification). At least 300 sperm were counted and classified as dead (pink, eosin-stained) or live (unstained).

The hypo-osmotic swelling test (HOST) was conducted to assess sperm plasma membrane integrity [18]. The HOST solution (0.735 g sodium citrate and 1.351 g fructose dissolved in 100 mL distilled water; osmolarity 190 mOsmol/kg) was prepared. For each treatment, 50 µL of thawed semen was mixed with 500 µL HOST solution, incubated at 37 °C for 40 min, and then examined microscopically (Olympus BH-2, Japan; 400× magnification). At least 200 sperm per sample were evaluated; those with swollen, coiled tails were classified as having intact plasma membranes.

### 2.6. Lipid Peroxidation

MDA concentration, an indicator of lipid peroxidation, was measured using the thiobarbituric acid (TBA) reaction [19]. Semen samples were standardized to 250 × 10^6^ sperm/mL (prepared by centrifuging approximately 6 mL of thawed semen to obtain a sperm pellet, from which 500 µL was used) and were incubated with 0.25 mL of 1 mM ascorbic acid (Sigma, A5960) and 0.25 mL of 0.2 mM ferrous sulfate (Ajax, 0906251, Ajax Finechem Pty Ltd., Wollongong, Australia) for 1 h at 37 °C. Subsequently, 1 mL of 15% trichloroacetic acid (Sigma, T6399) and 1 mL of 0.375% TBA (Sigma, T5500) were added. Samples were boiled for 10 min, cooled to 4 °C, centrifuged (4 °C, 4000× *g* for 10 min), and analyzed by UV–Vis spectrophotometry (Analytik Jena Specord 250 plus) at 532 nm. MDA concentrations are reported as µM/mL.

### 2.7. Statistical Analysis

Before conducting statistical analysis, the data were tested for normal distribution using the Shapiro–Wilk test, and homogeneity of variance was evaluated using Levene’s test. Data were analyzed using a randomized complete block design (CRD). Tukey’s post-hoc test was used to compare MDA levels and sperm quality parameters among the treatment groups. Differences were considered statistically significant at *p* < 0.05. Statistical analyses were performed using IBM SPSS Statistics (version 28.0; (SPSS Inc., Chicago, IL, USA)).

## 3. Results

### 3.1. TPC and Antioxidant Capacity of MOLE

The TPC of MOLE was determined to be 3.18 ± 0.03 mg GAE/g of dry weight. MOLE exhibited a DPPH radical scavenging activity of 67.60 ± 0.85%.

### 3.2. Fresh Semen Quality

The initial fresh semen samples from native Thai bulls had a mean volume of 5.97 ± 1.23 mL and a sperm concentration of 578 ± 2.75 × 10^6^ sperm/mL. Mean sperm motility was 87.65 ± 4.81%, while sperm viability was 91.22 ± 3.23%.

### 3.3. Effect of MOLE on Post-Thaw Semen Quality

The effects of various MOLE concentrations (0, 0.5, 1, and 1.5 mg/mL) on post-thaw semen parameters, including sperm motility, viability, and plasma membrane integrity are shown in Table 1. Post-thaw semen analysis revealed a significant concentration-dependent effect of MOLE on several key sperm parameters (*p* < 0.05). A MOLE concentration of 1 mg/mL consistently yielded favorable outcomes. This concentration significantly improved MOT, PMOT, viability, and plasma membrane integrity compared to the control and other MOLE concentrations. However, increasing the MOLE concentration to 1.5 mg/mL resulted in a negative effect on all sperm quality parameters compared to those in the other concentration groups and control (*p* < 0.05).

Data on the movement characteristics of sperm after thawing are presented in Table 2. At the optimal concentration of 1 mg/mL MOLE, VCL was significantly higher than the levels in all other groups (*p* < 0.05), indicating enhanced sperm movement. Similarly, ALH, a measure of sperm head oscillation reflecting motility vigor, was significantly greater at 1 mg/mL than in the control and other treatment groups (*p* < 0.05). Other motility parameters, including BCF, STR, and LIN, showed no significant differences across the MOLE concentrations tested.

**Figure 1:**
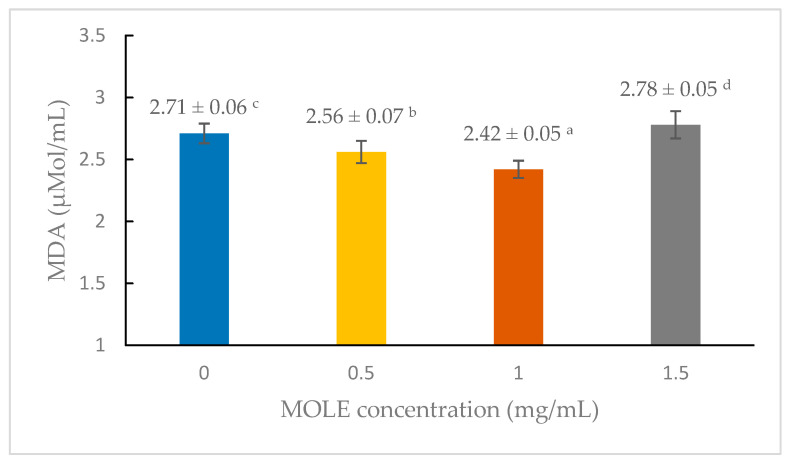
Effects of MOLE concentrations on lipid peroxidation of frozen–thawed semen. Values with different superscript letters ^(a, b, c, d)^ indicate significant differences between MOLE concentrations (*p* < 0.05).

Figure 1 illustrates the effects of different MOLE concentrations on lipid peroxidation (MDA levels) in post-thaw semen. No concentration-dependent effects on the MDA levels were observed. All MOLE concentrations (0.5, 1, and 1.5 mg/mL) displayed comparable MDA levels, with 1 mg/mL showing slightly lower MDA levels than the control.

## 4. Discussion

This study demonstrated that incorporating MOLE into a cryopreservation protocol significantly enhanced the post-thaw quality of semen from native Thai bulls. The most notable improvements were observed at a concentration of 1 mg/mL MOLE, which resulted in significantly higher MOT, PMOT, plasma membrane integrity, and viability values compared to those in the control and other treatment groups. These findings align with previous studies highlighting the benefits of antioxidant supplementation in improving cryopreserved sperm quality. For example, Lottus Mphaphathi et al. [20] observed improved sperm motility and membrane integrity in livestock species semen supplemented with antioxidant-rich plant extracts. Similarly, Georgieva [21] reported the potential benefits and mechanisms of action of herbal extracts as antioxidants in ram semen. These improvements suggest that MOLE supplementation has the potential for enhanced fertility in native Thai bulls, warranting further investigation through in vivo studies to assess pregnancy rates and reproductive efficiency.

The cryoprotective effects of MOLE were primarily attributed to its potent antioxidant properties. The antioxidant capabilities of MOLE are well-documented, with polyphenols and flavonoids playing a vital role in neutralizing ROS and mitigating oxidative damage during the freeze–thaw processes. In this study, TPC in the extract was measured at 3.18 ± 0.03 mg GAE/g, exhibiting significant free radical scavenging activity as evidenced by a DPPH inhibition of 67.60 ± 0.85%. These findings align with earlier studies highlighting the robust antioxidant capacity of *Moringa* extracts, which has been linked to improved cellular resilience under oxidative stress [22,23]. Consistent with these results, MOLE treatment significantly reduced MDA levels, a biomarker of oxidative stress, in cryopreserved sperm cells (Figure 1). This reduction was most pronounced at a concentration of 1 mg/mL, suggesting an optimal dose-dependent protective effect against lipid peroxidation. These findings align with previous research that demonstrated that the antioxidant activity of MOLE mitigates cryopreservation-induced oxidative stress. They also corroborate previous research demonstrating the efficacy of *Moringa* antioxidants in scavenging free radicals and reducing oxidative damage in various cell types [13]. For instance, Carrera-Chávez et al. [24] reported that *Moringa* extract reduced oxidative damage in cryopreserved ram semen through its free radical scavenging activity. Similarly, studies on cryopreserved buffalo and shrimp semen have underscored the ability of *Moringa*-derived antioxidants to maintain cellular integrity and improve post-thaw sperm viability [11,25]. However, a comparative analysis reveals variability in the optimal MOLE concentrations across studies and species. In goat semen, a concentration of 400 µg/mL yielded significant cryoprotective effects [9], while buffalo bull semen benefited most from supplementation at 600 µg/mL and 1500 µg/mL, as reported by Iqbal et al. (2022) and Shokry et al. (2024), respectively [10,11]. This variability likely reflects the influence of extender composition, sperm physiology, and cryopreservation protocols on the antioxidant requirements of sperm cells [26]. Such differences highlight the need for species-specific optimization of MOLE supplementation protocols to achieve maximal efficacy.

Although 1 mg/mL MOLE optimized MOT and PMOT, no significant differences were observed across MOLE concentrations for several other kinematic parameters (VAP, VSL, VCL, BCF, STR, and LIN; Table 2), indicating that MOLE has a selective effect on specific aspects of sperm function. Similar findings have been reported in other species, where antioxidant supplementation improved motility and viability but had a limited impact on specific kinematic parameters [26,27]. The selective effects observed in this study warrant further research to elucidate the precise mechanisms by which MOLE enhances sperm cryo-survival, potentially involving targeted interactions with sperm metabolic pathways or structural components.

The improvements observed in specific kinematic motility parameters provide additional insights into the effects of MOLE. The ALH at 1 mg/mL MOLE was significantly greater than that in the other groups, suggesting enhanced sperm head movement. A strong correlation between ALH and fertilization ability has been well-established in the literature, as increased ALH reflects improved flagellar activity and axoneme integrity [28]. In other words, ALH is widely regarded as a marker of sperm hyperactivation, a crucial physiological state required for successful fertilization. Furthermore, the observed increase in VCL suggests enhanced motility vigor, which is an essential factor in the sperm’s ability to penetrate the zona pellucid and achieve fertilization [29]. The combination of high ALH and VCL indicates robust mitochondrial function, as mitochondria provide the energy required for vigorous sperm motility. Importantly, these improvements in ALH and VCL are likely a result of MOLE’s broader preservation of the sperm’s kinetic apparatus and axoneme integrity rather than a direct and isolated effect on these specific parameters. This complex action underscores MOLE’s potential to maintain the structural and functional integrity of the sperm flagellum, enhancing its capacity to navigate the female reproductive tract and achieve fertilization [30].

Interestingly, the highest MOLE concentration (1.5 mg/mL) did not yield the best quality post-thaw semen. This observation coincides with a concomitant increase in MDA levels, suggesting that excessive antioxidant supplementation may disrupt the cellular redox balance, leading to pro-oxidant effects [31]. In this phenomenon, known as the “antioxidant paradox”, has been widely reported, high doses of antioxidants can impair ROS-scavenging mechanisms and result in oxidative damage [32,33]. Additionally, high concentrations of antioxidants have been linked to cytotoxic effects on sperm cells, as previously observed in studies involving excessive antioxidant supplementation in cryopreserved semen [34]. Similar negative outcomes of high antioxidant doses have been observed in various species. For instance, high levels of sericin in bull sperm extenders led to reduced post-thaw sperm quality, potentially due to an imbalance in oxidative stress regulation [26]. Similarly, excessive supplementation of other antioxidants, such as *aloe vera*, *ginseng*, and *Eurycoma longifolia* extracts, adversely affected sperm viability and motility in rooster sperm [16,35,36]. These findings highlight the importance of determining the optimal MOLE concentration to maximize the antioxidant benefits while minimizing the potential adverse effects. Maintaining a balanced approach is crucial for fully exploiting the cryoprotective properties of MOLE without triggering pro-oxidative responses.

## 5. Conclusions

This study explored the use of MOLE to improve the quality of freeze–thawed semen from native Thai bulls. Different concentrations of MOLE, rich in antioxidants, were added to a standard semen extender, and the semen samples were subsequently cryopreserved. The results demonstrated that the addition of 1 mg/mL MOLE to the semen extender significantly improved sperm motility viability and plasma membrane integrity after thawing. In contrast, higher or lower concentrations of MOLE did not produce the same beneficial effects. Additionally, MDA levels, a marker of oxidation-induced cell damage, were slightly reduced at 1 mg/mL MOLE concentration compared to the levels in the control group. These findings suggest that MOLE may be a valuable tool for improving the success of artificial insemination and breeding programs in native Thai bulls, warranting further in vivo studies to confirm its efficacy.

## Figures and Tables

**Table 1:** Effects of different MOLE concentrations on sperm motility (total and progressive motility), sperm viability, and plasma membrane integrity of frozen–thawed semen (mean ± SE).

Semen Parameter	MOLE Concentration (mg/mL)			
	0	0.5	1	1.5
MOT (%)	46.52 ± 0.77 ^c^	49.98 ± 0.76 ^b^	58.96 ± 0.81 ^a^	42.47 ± 0.92 ^d^
PMOT (%)	35.65 ± 0.59 ^c^	37.90 ± 0.56 ^b^	44.07 ± 0.82 ^a^	31.43 ± 0.94 ^d^
Viability (%)	53.07 ± 0.55 ^c^	56.07 ± 0.58 ^b^	61.76 ± 0.57 ^a^	50.51 ± 0.54 ^d^
Plasma membrane Integrity (%)	53.75 ± 0.58 ^c^	56.84 ± 0.56 ^b^	63.34 ± 0.63 ^a^	51.43 ± 0.61 ^d^

MOLE, *Moringa oleifera* leaf extract; MOT, percentage of total motility; PMOT, progressive motility; ^a, b, c, d^ different letters in the same row indicate statistical differences (*p* < 0.05).

**Table 2:** Post-thaw kinematic sperm motility parameters at various MOLE concentrations.

Kinematic Parameters	MOLE Concentration (mg/mL)			
	0	0.5	1	1.5
VAP (μm/s)	82.78 ± 0.07	83.08 ± 0.06	83.14 ± 0.06	83.01 ± 0.07
VSL (μm/s)	62.83 ± 0.05	62.84 ± 0.06	62.76 ± 0.04	62.59 ± 0.06
VCL (μm/s)	124.09 ± 0.39 ^c^	127.51 ± 0.41 ^b^	130.92 ± 0.40 ^a^	123.46 ± 0.37 ^c^
ALH (μm/s)	6.72 ± 0.03 ^bc^	6.81 ± 0.04 ^b^	7.30 ± 0.03 ^a^	6.65 ± 0.05 ^c^
BCF (Hz)	26.63 ± 0.06	26.64 ± 0.07	26.71 ± 0.05	26.68 ± 0.04
STR (%)	75.89 ± 0.15	76.11 ± 0.17	75.44 ± 0.12	75.42 ± 0.16
LIN (%)	50.24 ± 0.21	49.37 ± 0.23	49.80 ± 0.22	50.21 ± 0.20

MOLE, *Moringa oleifera* leaf extract; VAP, average pathway velocity; VSL, straight-line velocity; VCL, curvilinear velocity; ALH, amplitude of lateral head displacement; BCF, beat cross frequency; STR, straightness (VSL/VAP); LIN, linearity (VCL/VAP); ^a, b, c^, different letters in the same row indicate statistical differences (*p* < 0.05).

## Data Availability

The data are available upon request from the corresponding author.
